# Navigating knowledge landscapes: on health, science, communication, media, and society

**DOI:** 10.3325/cmj.2015.56.321

**Published:** 2015-08

**Authors:** Anna Lydia Svalastog, Joachim Allgaier, Srećko Gajović

**Affiliations:** 1 Psychosocial Work, Department for Health and Social Studies, Østfold University College, Halden, Norway; 2 Centre for Research Ethics and Bioethics, Uppsala University, Uppsala, Sweden; 3 Alpen-Adria-University, Institute of Science, Technology and Society Studies, Klagenfurt, Austria; 4 University of Zagreb School of Medicine, Croatian Institute for Brain Research, Zagreb, Croatia

Today patients are confronted with an increase in complexity of health-related information, including that on medical procedures, data interpretation, and multifaceted therapeutic strategies. At the same time, there is a justified need to simplify information in order to enable patients to make decisions about themselves (1). The patient is indeed the only one in the system who possesses all the information and insights into her/his health and biomedical biography. Health related issues are important not only to patients, but to all citizens who take health into consideration when making everyday lifestyle decisions, such as choice of diet, or physical or social activities. As we live in a society full of opportunities, navigating wisely through them, and making educated decisions, clearly requires more steps than just declaratively empowering the patient by the medical system. An important prerequisite for patient-centered medicine is ensuring that patients can find and make use of high-quality knowledge about science and biomedicine.

If we want to interpret the relevance and meaning of information in a particular situation we require knowledge. The access to traditional or new knowledge was previously based on institutionalized expertise. Societal institutions and cultural understandings framed the dissemination of knowledge and stories on health related issues (2). Still in the present globalized and digitalized society, information and knowledge is distributed freely in ways that blur the relation between institutionalized expertise and more general information. In addition, the accumulation and distribution of knowledge, by experts and lay people, are interwoven with economic relations, legal, and administrative regulations.

Communication is an essential tool for establishing good psychosocial conditions for the user, the professionals, and people in general. The landscapes of communication in present society are complex, interacting, and overlapping. A person searching for or being exposed to medical advice online regarding an outbreak (epidemic or pandemic), a common complex disease (Alzheimer, diabetes, cancer, cardiovascular diseases), social or mental health issues, addiction, or substance abuse is targeted by a variety of senders. Information is accessible from different sources, including contact information, institutional domains, research news, personal stories, rumors, or critique. The senders represent various backgrounds and motivations, including interest groups, serious participants, but also insincere actors. Therefore, the new ways of how and what information is distributed, and how it affects individuals’ decisions need to be investigated and understood.

To address these issues we have initiated the Knowledge Landscapes collaborative group. Its aim is to develop an international and interdisciplinary collaboration, which will explore the area of online and offline communication and distribution of medical information with impact on treatment, recovery, well-being, and quality of life. We believe that a key to empowering the patient and assuring the quality of life in general lies in the trustworthy communication of and about biomedical and scientific knowledge. The improved communication needs to be at the center of the future medicine (3). As a way to communicate the results of this collaborative and interdisciplinary endeavor, Knowledge Landscapes will be a new headline topic for an essay series in the *Croatian Medical Journal* (4).

## The challenges of Knowledge Landscapes

Technological and digital innovations represent particular challenges for users and distributors of medical and health related knowledge and practices, but also for those governing and regulating the exchange of knowledge online. So far there have been no global guidelines on how to regulate various health information and services offered on the internet, which often opened serious ethical questions (2). Topics of concern are many: advertising treatments related to common complex diseases, accessibility of genetic information, bio bank issues, stem cell research and therapies, globalized risks such as pandemics, public health strategies like vaccination.

Knowledge communication between different stakeholders is deeply affected by new online tools like internet homepages of medical and research institutions, patient interest groups, video-sharing websites, social media and blogs, digitalized health records and other registers, mobile devices and smartphones, etc. User are in the same time directly interacting with the online resource and are physically distanced from it, as it could be located anywhere in the world. They can remain anonymous and do their reading from situations and interpretative contexts that can differ substantially from those of the intended receiver of information. The complex interactions between technology, knowledge, social contexts, stakeholders and their various agendas, policy, economy, and worldviews represent a future challenge for interdisciplinary elaborations.

## What are Knowledge Landscapes?

The one-way (donor to recipient) communication strategy of knowledge is not ideal, simply because it does not allow dialogic exchange of knowledge and information. Government agencies still use one-way communication strategies, despite new communication technology environments (5). One-way communication is problematic because it does not allow feedback, questions, clarifications, and does not allow verification or correction. Therefore, we think that dialogues or even multi-logues are necessary to advance communication about knowledge. The Knowledge Landscapes blueprint is a concept under development, which uses an analogy to three dimensional space to represent multi-directional knowledge communication among many participants using modern and traditional technologies (2). Three dimensional communication landscape includes many participating stakeholders and information visible and shared among them.

Our hypothesis is that the development of new technologies will improve the communication and exchange of knowledge in various knowledge landscapes. Technologies enabling the communication in multiple directions are expected to provide better accuracy and higher relevance of the exchanged knowledge. Consequently, if we understand the precise needs for the use of such technologies, they can be envisaged and developed. In this way, we hope that the Knowledge Landscapes concept will be helpful for the navigation through various landscapes and forms of knowledge with fewer misunderstandings, disagreements, distortions, and misinformation. However, we cannot rely on technology alone: technological advances can as well have unintended side effects and distort knowledge communication.

## Empirical analysis and critical methodology

The example of topics related to Knowledge Landscapes can entail knowledge contents on the internet vs knowledge stored offline, the role of smartphones, or digitalized journals. Knowledge platforms and tools that are not available online are not excluded from the analysis. For example, two of the authors (JA and AS) are currently looking into the case how information and misinformation about Ebola has spread during the outbreak. The communication and dissemination of rumors via mobile phones influenced treatment or non-treatment of Ebola infections.

In the offline world, it is common that knowledge is exchanged among many participants, and three dimensional communication models and spaces have already been discussed in various disciplines long before the internet era. However, online and digital technologies pose entirely new sets of questions and problems. For instance, search engines and personalization of internet searches do not only help us to find information, they can also hide knowledge from us or make it much more difficult to find particular knowledge, thereby creating what we call back holes of knowledge and information (6). At the same time, some providers, knowledge, and information are privileged through search engines. Rogers (7) describes them as having “algorithmic authority” – thereby influencing which links are considered more relevant and important. Furthermore, personalized searches are influenced by own search histories and this may create the effect of an echo chamber of information that always finds “more of the same.”

We want to stress that the online world and social media introduce new channels and new obstacles affecting Knowledge Landscapes (8). We are interested in the ways how this new situation affects the content and functions of communication and social life. Are we about to see a new social organization of knowledge or more distorted and harmful information being distributed? How do we implement ethics (ethics in general, research ethics, clinical ethics etc.) in these new circumstances (9)?

If one-way communication strategies are a bad way of distributing information, how do we initiate discussions on conflicting issues, which could lead to understanding but also disagreement? How should we deal with harmful, biased, erroneous, and distorted knowledge about health and medicine that patients find browsing the web? And how should we deal with the increasing amount of data and “big data” analysis (10) that will inevitably also find its way into biomedicine (11)?

The aim of the Knowledge Landscapes analyses is to facilitate navigation through knowledge with fewer misunderstandings, disagreements, distortions and misinformation. Patients should be empowered to be rightly at the center of their health care, based on a better understanding of biomedicine, communication media, and technology. But Knowledge Landscapes have even wider implications, as everybody shall be in the center of activities relevant to their own life, rendering knowledge management essential for our democratic society.
